# Workplace wellness: industry associations are well placed and some are ready to take a more active role in workplace health

**DOI:** 10.1186/s12913-018-3364-7

**Published:** 2018-07-18

**Authors:** Geraldine Marsh, Virginia Lewis, Jenny Macmillan, Su Gruszin

**Affiliations:** La Trobe University, Melbourne, VIC Australia

**Keywords:** Industry associations, Workplace health promotion programs, Workplace wellness

## Abstract

**Background:**

Investments in settings-based health interventions can include workplaces, however, engaging with businesses and convincing them to take a role can be difficult. Our research investigated the potential for trade or industry associations (IAs) to have a role in promoting workplace health initiatives to their members.

**Methods:**

Seventeen semi-structured interviews were undertaken with senior executives from IAs representing industries in the mining, transport, agriculture, manufacturing, farming, hospitality, and construction sectors. Analysis of interviews identified themes around attitudes to workplace health promotion programs and the perceived, actual and potential role/s of IAs in promoting workplace wellness.

**Results:**

IA representatives believed workplaces had potential to be promoting the health and wellbeing of workers through their member organisations; however for some the extent of their role was unclear and for others there was confusion between government-mandated safety initiatives and non-mandated health and wellbeing initiatives. All reported that their IA could have a role in promoting worker health and wellbeing initiatives to member organisations. IAs with larger companies as members were more likely to recognise the importance of workplaces promoting workers’ health; however, the degree of involvement considered appropriate varied. Most IAs had not discussed the topic with their member organisations although they identified resources and support that could assist them in encouraging members to undertake workplace health programs. Resources included industry-relevant business cases outlining the benefits of workplace health, and industry-appropriate worker health information.

**Conclusions:**

Our research suggests that across many industry sectors, larger IAs in particular are ready to take a more active role in workplace health initiatives and are well placed to promote these to member organisations.

**Electronic supplementary material:**

The online version of this article (10.1186/s12913-018-3364-7) contains supplementary material, which is available to authorized users.

## Background

The potential of workplaces to be sites for investment in setting-based health promotion is recognised particularly in countries such as the United States where there are financial incentives through health insurance schemes for employers to take an active role in their employees’ health and wellbeing. Even where such incentives are absent there is still evidence for the benefits to workplaces in promoting workers’ health and wellbeing including improved morale, reduced absenteeism and increased levels of productivity [1–7]. Carmichael and colleagues reviewed the evidence for such programs and their impact on individuals and workplaces across a range of industries [4]. They concluded that preoccupations with workplace health and wellbeing programs could be explained by a mix of business norms of responsibility towards employees and belief in the positive impact of employee health and wellbeing on business productivity and performance [4]. In spite of this, not all businesses provide health promotion initiatives in their workplaces. It is a challenge to convince these businesses to take up a health promotion role.

Research into workplace health promotion programs has tended to focus on the benefits of such programs and program design, including incentives to implement programs. There is little research on strategies to increase employer up take of such programs outside direct funding and other incentives.

Our research investigated the views of industry associations (IAs) on workplace health promotion and whether they had a potential role in promoting workplace wellness programs. ‘Industry association’ (or peak industry body or council in Australia) is a term used for an advocacy group or an association of industries or trades with allied interests; member organisations are usually organisations or companies that employ workers. The roles of IAs, acting for the collective needs and objectives of their members, can include: influencing government policy, and regulation; influencing public opinion; disseminating and exchanging information within industries; and acting as informal regulators by setting voluntary standards of behaviour for members [8]. In the related occupational health and safety (OH&S) field, although most policy and legislation relates to large enterprises, IAs have been noted as able to influence the improvement trajectory and regulatory compliance in small and medium-sized enterprises [9, 10]. Despite the significance of their roles, IAs are relatively poorly studied [8].

‘Workplace wellness programs’ includes any workplace health promotion activity or organizational policy designed to support healthy behaviour in the workplace and to improve health outcomes. Examples include Quit smoking and physical fitness programs, and healthy eating promotions such as provision of fresh fruit in work canteens.

This study was conducted as part of an evaluation of an active workplace health and wellbeing program implemented through the Queensland state government [11]. Queensland is the second-largest state in Australia, and the third-most-populous, with a diversified economy that includes agricultural, resource, construction, tourism, manufacturing and service sectors [12, 13]. In particular we explored whether IAs are, or could act as, ‘champions’ to promote the role of workplaces in diverse industries in supporting the health and wellbeing of their workers [14, 15].

## Methods

As part of our evaluation of a state-wide initiative across Queensland, Australia, where a workplace health and wellbeing program was being implemented through the state government [11], we investigated the views of senior executives from IAs across the major industry groups in the state. A semi-structured interview schedule was constructed with input from relevant state government workforce health and safety experts, who also assisted in identifying appropriate IAs through a web scan of representative employer bodies and member organisations (Additional file 1).

Seventeen semi-structured interviews were conducted with senior executives from IAs representing the mining, transport, agriculture, manufacturing, farming, hospitality, and construction sectors. When organising interviews, efforts were made to ensure that the participant was in a position that was, or would be, responsible for health and wellbeing policy and practice in the organisation. An inductive content analysis was used to identify themes [16] around attitudes to workplace health promotion programs and the perceived, actual and potential role/s of IAs in promoting workplace wellness. The data were initially analysed by one member of the research team (GM), with emerging themes subsequently confirmed through independent analysis, and discussion among other members of the research team (JM, VL).

## Results

All of the senior executives of IAs who were interviewed believed that workplaces had a role in promoting the health and wellbeing of their workers. Some, however, were unsure what the role was, and some thought the role was limited. The latter was particularly the case where the IA’s member companies/businesses were small and had limited capacity to resource such a role. It was also the case where there was a perception that health and wellbeing were personal rather than employer responsibilities, especially in sectors such as agriculture or hospitality with a higher proportion of transient and casual workers, or where there is a high level of subcontracting. IAs in sectors that relied on a high level of expertise and that potentially had difficulty recruiting (or retaining) workers reported higher levels of interest in health and wellbeing programs for workers.

A number of those interviewed confused government-mandated occupational health and safety (OH&S) requirements that emphasise workplace safety, with health and wellbeing programs which are optional and use the workplace as a setting for health promotion activities. Representatives from IAs with larger, national or international companies as members, or those with previous involvement in health and wellbeing programs, were more likely to distinguish between these programs and safety requirements.

All of the executives interviewed believed that their IA could have a role in promoting the importance of worker health and wellbeing to their member organisations, however the degree of involvement considered appropriate varied, with IAs whose members were larger companies more likely to recognise the importance of workplaces in promoting workers’ health. Most of the senior executives interviewed recognised that members could benefit from workplace health and wellbeing programs, however they reported that their IAs had not discussed the topic with their members. Some commented that their role was to reflect the interests of their member organisations rather than direct them, and therefore they would only take on a role if there was an expectation from their members to do so. Others expressed concern at the potential cost to their member organisations relative to the perceived benefit.

As noted above, there was some confusion between government-mandated OH&S requirements and workplace health and wellbeing programs. Senior executives were clearer about the assistance they could provide around safety compliance. When asked to give examples of how they could or did support member organisations in promoting the health and wellbeing of their workers, the majority responded with examples around safety such as chemical storage, safe tractor driving, safety clothing or health issues arising from poor safety.

Senior executives did, however, identify resources and support that would assist them in encouraging their member organisations to undertake workplace health programs. These focussed on information and resources including the evidence of benefits, and access to services. Industry-relevant business cases outlining the benefits of workplace health, particularly the potential to improve productivity and therefore the financial return to businesses, were seen to be essential to encouraging the take up of programs. Industry-appropriate worker health information was also reported to be important, together with access to an advisor who could answer member organisations’ questions and assist with implementation of programs.

## Discussion

There are significant concerns around the rising costs of health care associated with ageing populations and increased incidence of chronic diseases and conditions [17, 18]. Although the current Australian government policy focus is on containing the costs of hospitalisation and clinical management rather than on prevention and health promotion, in the longer term prevention and health promotion are the essential components needed to address preventable hospital admissions [17].

Work has become more sedentary contributing to the increased incidence of obesity, and musculoskeletal and other health issues [5, 7, 19–25]. These potentially impact on workplace productivity and are key reasons for employers to initiate workplace health promotion programs. In some sectors such as mining and construction, workers may live on-site for at least some of the time, especially when jobs are in remote areas. In these cases in particular, it could be argued that the employer has a responsibility to ensure that there are opportunities for employees to engage in healthy lifestyles.

Workplaces can provide opportunities for group activities and incentives to participate in health promotion that may not be available elsewhere. There is now a body of evidence that workplace health and wellbeing programs benefit both the individuals participating as well as the businesses for which they work [1–7]. Challenges remain, however, with involving individual businesses in workplace health and wellbeing programs.

As noted above, the IA senior executives interviewed indicated some confusion between government-mandated OH&S requirements (e.g. legislated requirements for workers in some industries to wear safety clothing) and workplace health and wellbeing programs (e.g. promoting Quit smoking and physical fitness programs in which workers can choose to participate or not). This may be particularly the case when the latter programs are being promoted by government which also monitors compliance with occupational safety legislation. Our research indicated that this confusion is shared by both IAs and their member organisations. Although confusion may not on the surface appear to be an issue, it could skew the data around uptake of programs. For example, when asked: does your organisation have a role in workplace health and wellbeing? some may respond in the affirmative when in fact they are involved in meeting the requirements of OH&S regulations rather than providing workplace health promotion activities. The confusion may also discourage health promotion program uptake, as organisations may feel they are already doing something and be reluctant to do more.

Reach is also an issue. When advocating for and supporting delivery of health and wellbeing programs, approaches to individual organisations may not be the most efficient or cost-effective approach for government. Although IAs may need to be provided with resources, they are likely to be a more cost-effective way to reach and support uptake of programs in hard-to-reach locations. The costs of introducing health and wellbeing programs can also be a barrier for workplaces, particularly small ones. In the Danish experience related by Kvorning and colleagues, regulators and other stakeholders including IAs, developed programs with financial and facilitator support to assist small enterprises with limited resources [26]. Credibility may also be an issue especially if programs are being promoted by government to the business sector [8, 26, 27]. Program credibility can be built and advocacy for programs can be delivered across large numbers of workplaces using the existing networks and capacity of IAs. IAs are therefore well placed to act as intermediaries between governments wanting program implementation and those directly involved ‘at the coalface’ [26].

Carmichael and colleagues summarised the necessity, before developing an intervention, of understanding the characteristics of the organisation, its work practices, work environment, policies and the workforce (including attributes such as health status and fitness for tasks, beliefs and perceptions) [4]. Through IAs, and their understanding of their member organisations, the language and appropriateness of promotional materials and resources can be refined. As one interviewee stated:

Mates in Construction [a construction industry health program] works because it is presented in the language and approach guys on the worksite understand, workers are positive about the program and it is relevant to them.

A basic mechanism for developing best practice is the provision of opportunities for like organisations working on similar initiatives to share knowledge and experiences [18, 28, 29]. IAs provide an excellent opportunity for doing this through their existing networks and processes such as member newsletters and forums. While outside experts can be brought in to provide additional perspectives, the IAs are likely to provide greater credibility and a more trusted space for discussion [15, 24, 27]. As a type of ‘intermediating organisation’, IAs can also coordinate the activities of would-be or early adopters, and shape the diffusion trajectory of commercial innovations [30].

### Strengths and limitations

Themes presented in this investigation are specific to the 17 participating senior executives representing their IAs, and as such only those who were willing to discuss their views are included. There may have been other important and/or alternative views that were not canvassed. The senior executives who agreed to provide their views may have been more interested in workplace health and wellbeing than others who did not provide their views. Our findings may also have limited generalisability due to the small and varied sample. The views provided, however, represent a diversity of positions, drawn from senior executives in a range of IA types from the very small to the large and well resourced, and include broad coverage of the economically important industries in the state.

## Conclusions

Our research suggests that Industry Associations (IAs) have been overlooked as an agent for change, and that many are ready to take a more active role in workplace health promotion, particularly those representing larger member organisations. Policy initiatives and programs that seek to encourage workplace health promotion programs could be strengthened by involving IAs in all steps from program formulation through to the implementation of programs in workplaces.

Theoretically, IAs are well placed to extend workplace health promotion program reach and effectiveness in the large numbers of workplaces that are their members and in many industries. IAs understand the specific circumstances, needs and challenges experienced by the industries they represent. They can provide credible arguments on the relevance of health promotion within their sectors, and they have the networks and in many cases the resources to work with government regulators, employers and workplaces to extend the reach of initiatives and influence change.

Additional research into the differences between IAs depending on their size (in particular) and the workforce attributes of their member organisations may be useful. Key differences identified in our research related to the size of member organisations, and the type and employment arrangements of workers. For example: (a) larger IAs were more likely to recognise the importance of workplaces in the role of promoting workers’ health; and (b) those with smaller, more limited capacity member organisations (or in sectors such as agriculture or hospitality with a higher proportion of transient or casual workers or of subcontracting) were more likely to be unsure of, or think that their role should be limited. Further research could also identify the specific barriers that apply; for instance, lack of time and skill - directly related to small size - that IAs may need assistance to address.

The importance of worker health and well-being should be promoted to IAs in order for IAs to promote it in turn to their member organisations. Appropriate resources can be provided by relevant government departments and regulators, and non-government organisations (such as respiratory health fora and heart and stroke foundations). Opportunities to share knowledge and experiences among existing IAs and their member organisations should also contribute to the development and furthering of best practice in workplace health promotion programs. Many IAs are already well positioned to act as ‘champions’ in supporting and assisting their member workplaces to promote the health and wellbeing of their workers and the active involvement of IAs should be encouraged.

## Additional file

Additional file 1: Interview Guide, The Role of Peak Industry Bodies/Member Organisations Promoting the Health of Workers, four questions used to direct discussions. (DOCX 19 kb)

## Abbreviations

IAs: Industry Associations
OH&S: Occupational Health and Safety
