# Supplementary material for: Workplace wellness: industry associations are well placed and some are ready to take a more active role in workplace health
Source: BMC Health Serv Res. 2018 Jul 18;18:565. doi: 10.1186/s12913-018-3364-7 (PMC6052516; doi:10.1186/s12913-018-3364-7)
Supplement: Supplementary file 1 — Interview Guide, The Role of Peak Industry Bodies/Member Organisations Promoting the Health of Workers, four questions used to direct discussions. (DOCX 19 kb) [file 12913_2018_3364_MOESM1_ESM.docx]

**Additional File 1: Interview Guide: The Role of Peak Industry Bodies/Member Organisations Promoting the Health of Workers.**

1. **What role do you think workplaces should have in promoting the health and wellbeing of their workers?** (*Interviewer to explore:* Does it go beyond their Health and Safety legislative responsibilities?)

(*Prompts if required*: e.g. supporting workers to quit smoking, lunchroom encourages preparation of healthy food with fridge and microwave; chilled water tap; showers and lockers; stand-sit desks; walking meetings encourage reduce alcohol intake and exercise, link them to health services, )

*If yes…there is a role* **–** **Why (should they have an active role)? What kind of role?**

*Follow-up prompts:* What does it look like if a workplace does take on this role? What should they do - Can you provide any examples of workplaces that are promoting the health and wellbeing of their workers?

***If no…****shouldn’t have role –* **Why not?**

1. **Do you think your organisation has a role in supporting your members to promote the health and wellbeing of their workers?**

*If yes, follow-up questions:*

- **Why does your organisation sees it as part of their role?**
- **What does your organisation do to support members promote the health and wellbeing of their workers?** (*Possible* *Prompts*- provide health information, links to health web sites, links to health services, provision of example policies, system to encourage workplaces to promote the health of their workers promoting business benefits: good health = good business outcomes)
- **What have been the benefits of this engagement…**
  - **For your organisation?**
  - **For your members?**

*If no, follow-up questions:*

- **Why not?**
- **Is it something your organisation has considered doing?**

*If yes,*

**Are there barriers to you promoting the health and wellbeing of workers to your members?**

**What would assist them overcome these barriers?**

1. *FOR THOSE WHO SAY IT IS NOT THE ROLE OF WORKPLACE OR PIBS AND HAVE NOT PROVIDED A RESPONSE TO THE FOLLOWING IN THEIR PREVIOUS ANSWERS, ASK:* **Is there an organisation whose role it would be to promote general health and wellbeing in workplaces?**
2. **What role if any does the STATE Government have in supporting PIBS to engage with their members to promote the health of workers?**

(*Follow-up)*

- *If a role identified –* **What can Government do to support PIBs?**
- *If no role* – **Why not? Is there other support required?**

Thank you for your time
